# Differentiation of cancer stem cells into erythroblasts in the presence of CoCl_2_

**DOI:** 10.1038/s41598-021-03298-5

**Published:** 2021-12-14

**Authors:** Kazuki Kumon, Said M. Afify, Ghmkin Hassan, Shunsuke Ueno, Sadia Monzur, Hend M. Nawara, Hagar A. Abu Quora, Mona Sheta, Yanning Xu, Xiaoying Fu, Maram H. Zahra, Akimasa Seno, Masaharu Seno

**Affiliations:** 1 Department of Biotechnology and Drug Discovery, Graduate School of Interdisciplinary Science and Engineering in Health Systems, Okayama University, Okayama, 700-8530 Japan; 2 Division of Biochemistry, Chemistry Department, Faculty of Science, Menoufia University, Shebin El Koum, 32511 Egypt; 3 Department of Microbiology and Biochemistry, Faculty of Pharmacy, Damascus University, Damascus, 10769 Syria; 4 Department of Pathology, Tianjin Central Hospital of Gynecology Obstetrics, Nankai University Affiliated Maternity Hospital, Tianjin Key Laboratory of Human Development and Reproductive Regulation, Tianjin, 300100 China; 5 Department of Pathology, Tianjin University of Traditional Chinese Medicine, Tianjin, 300193 China

**Keywords:** Cancer, Cell biology, Stem cells

## Abstract

Cancer stem cells (CSCs) are subpopulations in the malignant tumors that show self-renewal and multilineage differentiation into tumor microenvironment components that drive tumor growth and heterogeneity. In previous studies, our group succeeded in producing a CSC model by treating mouse induced pluripotent stem cells. In the current study, we investigated the potential of CSC differentiation into blood cells under chemical hypoxic conditions using CoCl_2_. CSCs and miPS-LLCcm cells were cultured for 1 to 7 days in the presence of CoCl_2_, and the expression of VEGFR1/2, Runx1, c-kit, CD31, CD34, and TER-119 was assessed by RT-qPCR, Western blotting and flow cytometry together with Wright-Giemsa staining and immunocytochemistry. CoCl_2_ induced significant accumulation of HIF-1α changing the morphology of miPS-LLCcm cells while the morphological change was apparently not related to differentiation. The expression of VEGFR2 and CD31 was suppressed while Runx1 expression was upregulated. The population with hematopoietic markers CD34^+^ and c-kit^+^ was immunologically detected in the presence of CoCl_2_. Additionally, high expression of CD34 and, a marker for erythroblasts, TER-119, was observed. Therefore, CSCs were suggested to differentiate into erythroblasts and erythrocytes under hypoxia. This differentiation potential of CSCs could provide new insight into the tumor microenvironment elucidating tumor heterogenicity.

## Introduction

Cancer is one of the leading causes of death in Japan^1,2^. Due to the complicated difference between cancer patients, personalized treatment is important for effective therapy. Cancer stem cells (CSCs), also known as cancer-initiating cells, are one of the main foci for the development of therapeutic strategies. Within tumor tissues, CSCs have the potential to renew by themselves and to produce their progenies of cancer cells and associated cells critically controlling the nature of cancer diseases^3^. In addition, CSCs are believed to exhibit chemo-/radioresistance^4^, demonstrating recurrence and metastasis^5–7^. Hence, CSCs are currently attracting increased attention as a new therapeutic target in cancer treatment and are studied to identify their roles in the development of cancer at the molecular level.

However, the sources of CSCs are limited to cancer patients or animal models, and thus, the study of CSCs has ethical issues. We attempted to experimentally produce CSCs from stem cells, including induced pluripotent stem cells (iPSCs), and have been studying the characteristics of CSCs, such as pluripotency, self-renewal and tumorigenicity. Previously, most of our unique CSC models were developed by treating iPSCs with culture conditioned media (CM) of cancer derived cells^8–13^. During the time course, the CSC model was shown to differentiate into CD31-positive vascular endothelial cells^14^. In this report, vascular endothelial progenitor cells derived from CSCs were thought to be present in CD34^+^ cells and contribute to tumor angiogenesis. Moreover, another study revealed that CSCs differentiated into progenies with the morphology of hematopoietic precursor cells with specific markers and showed the ability to home to bone marrow^15^. These observations could provide insight into the various potentials of CSC differentiation depending on the stage of stemness.

However, changes in cellular characteristics under hypoxic conditions, which induce cytoplasmic responses mediated by hypoxia-inducing factor (HIF-1α), have been described more often than ever^16^. Generally, HIF-1α is degraded by ubiquitination under normal concentrations of oxygen, but degradation is inhibited to promote gene expression as a transcription factor when the oxygen concentration becomes low. Many reports have linked hypoxia with hematopoietic stem cell differentiation^17–20^. Similar results could be expected based on with CSCs differentiating into vascular endothelial cells as described above. If this is true, CSCs are expected not only to exhibit hematopoietic stem cell properties under hypoxic conditions but also to provide opportunities to differentiate into cancer-associated hematopoietic cells and to help elucidate the mechanism of acquisition of metastatic potential.

In the present study, we studied the ability of our CSC model to differentiate into blood type cells in the presence of CoCl_2_.

## Results

### Cobalt induces HIF-1α signaling and morphological changes in CSCs in vitro

**Figure 1:**
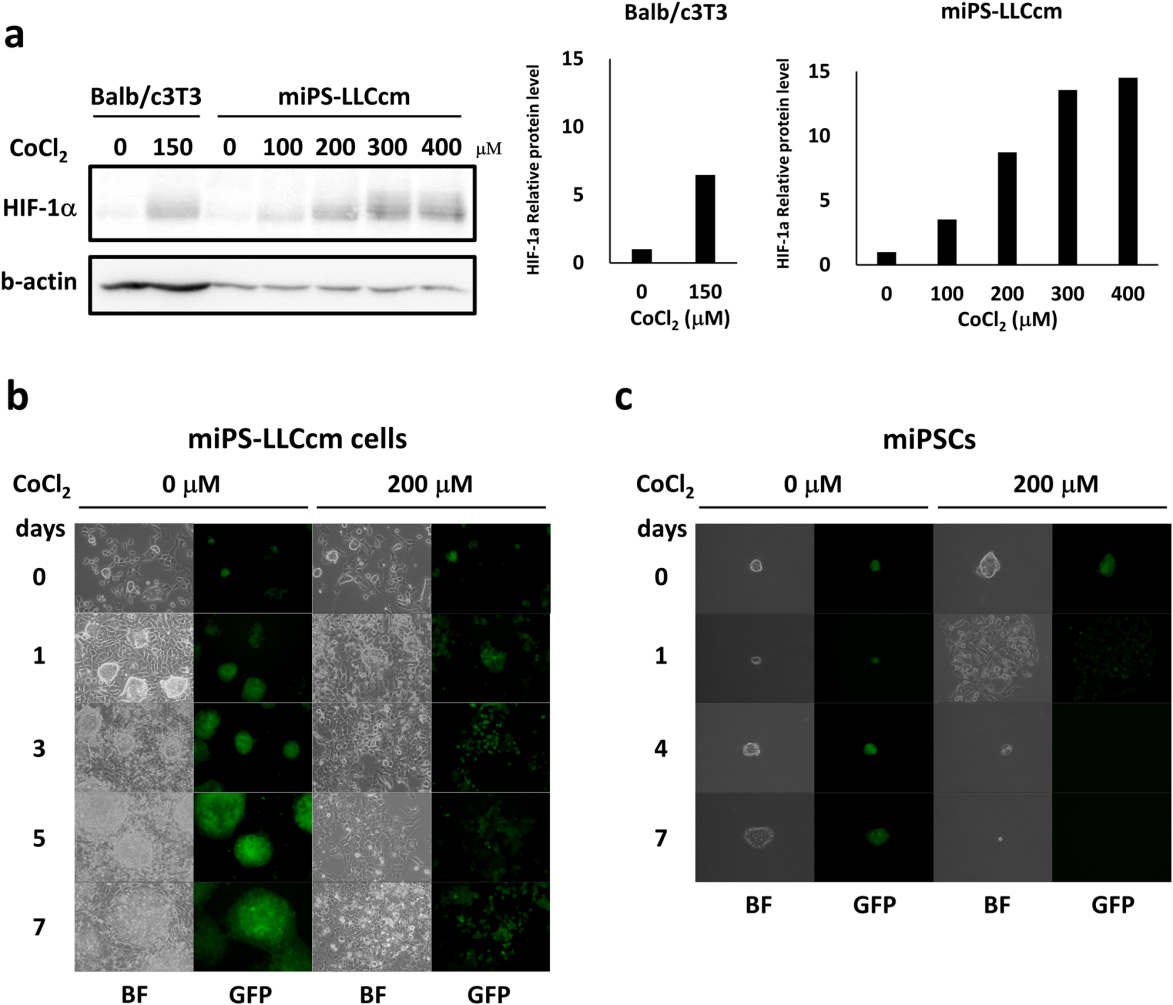
Effects of CoCl_2_ on CSC model miPS-LLCcm cells. (**a**) The increase in hypoxia-inducing factor HIF-1α in miPS-LLCcm cells in the presence of CoCl_2_ in the range of 0 to 400 µM was detected by Western blotting (left). The relative densitometric quantification was performed by ImageJ (right). Balb/c3T3 cells were used as a reference for the positive control. (**b**) The cell morphology and GFP fluorescence of miPS-LLCcm cells affected by 200 µM CoCl_2_ for 7 days. (**c**) The growth of miPS cells was significantly inhibited, and the cells differentiated or died during 7 days in the presence of 200 µM CoCl_2_ . (**b**, **c**) BF, bright field. GFP, fluorescence of 530 nm. Objective lens, × 20.

Previously, our group described the development of CSCs from mouse induced pluripotent stem cells (miPSCs) using the culture supernatant of the Lewis lung cancer cell line for 4 weeks^8^. The CSCs obtained was designated as miPS-LLCcm cells, which exhibited the differentiation potential to CD31^+^ vascular endothelial cells on Matrigel^14^. In the current study, the effect of CoCl_2_ on miPS-LLCcm cells was evaluated since Co^2^^+^ inactivates prolyl hydroxylases, which degrade HIF proteins depending on O_2_ concentration, replacing with Fe^2^^+^ to stabilize HIF just like in a hypoxia model^21,22^. CoCl_2_ was added in the culture of miPS-LLCcm cells for 1 to 7 days. During the time course, the fluorescence of green fluorescent protein (GFP), whose expression was controlled under the Nanog promoter, was monitored to identify undifferentiated subpopulations under a microscope. As a result, CoCl_2_ significantly elevated the expression of HIF-1α in miPS-LLCcm cells as well as in Balb/c 3T3 cells as a positive control (Fig. 1a) indicating the induction of HIF-1α signaling. Moreover, CoCl_2_ affected the morphology of miPS-LLCcm cells by suppressing the formation of colonies expressing GFP, while adhesive cells with and without GFP were easily distinguished from untreated miPS-LLCcm cells , which showed two subpopulations of cells, namely, large colonies expressing GFP and surrounding fibroblast like cells without GFP. These GFP-positive undifferentiated colonies enlarged according to the time of incubation. Under this condition, mouse iPS cells, which are the original cells used to be converted into CSCs, did not survive after Day 3 (Fig. 1b).

### Cobalt alters the differentiation potential of CSCs

**Figure 2:**
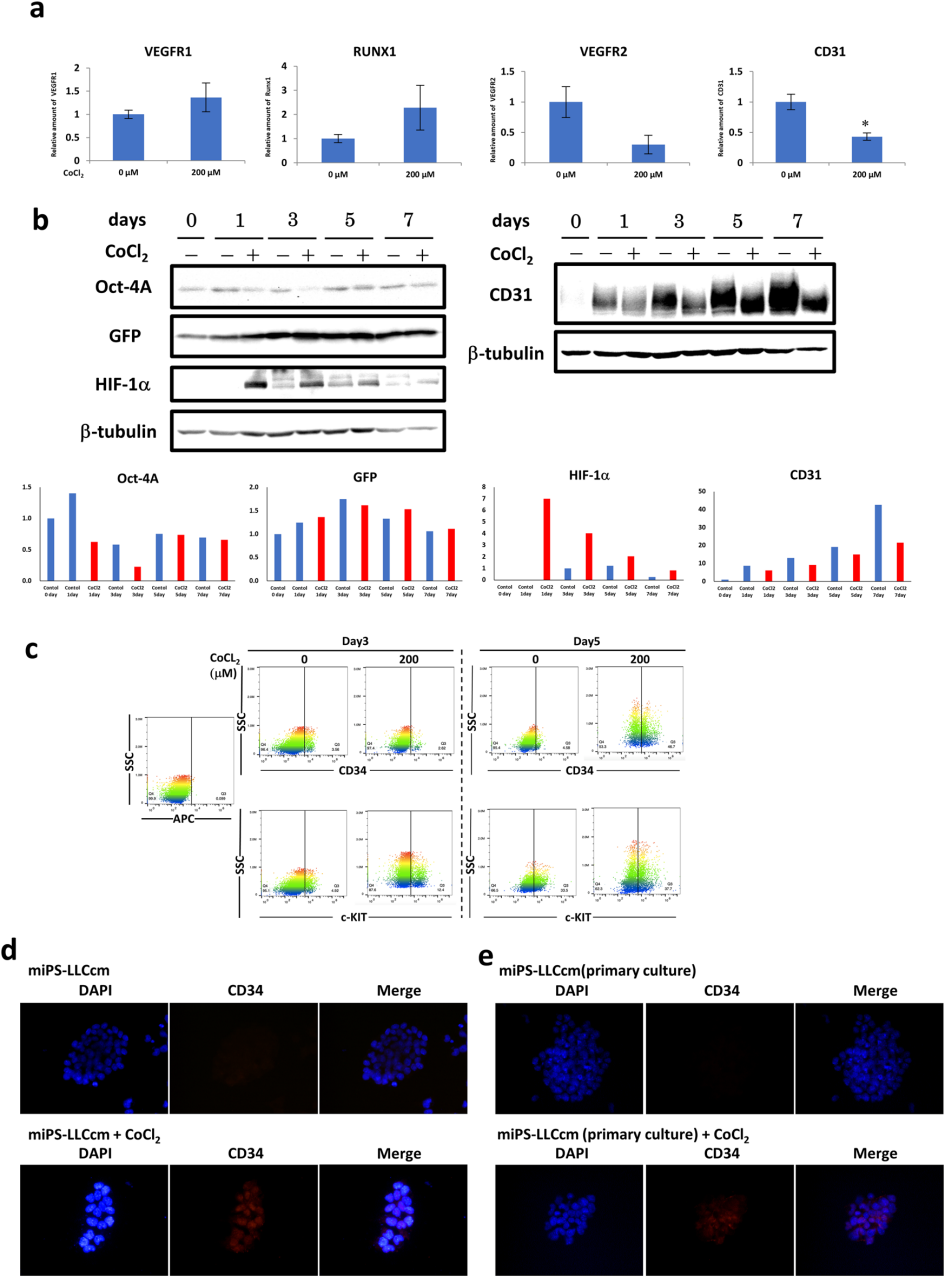
Evaluation of the differentiation of miPS-LLCcm cells in the presence of CoCl_2_. (**a**) The expression of hematopoietic markers induced in the presence of CoCl_2_. The expression of each gene was analyzed by RT-qPCR. The data were statistically analyzed by Student’s t-test. “*” indicates P < 0.05. (**b**) Western blotting analysis of Oct-4A, GFP, HIF-1α and CD31 over 7 days (top). The relative densitometric quantification was performed by ImageJ (bottom). (**c**) Flow cytometric analysis of CD34 and c-KIT positive populations vs. side scatter (SSC) at Days 3 and 5. (**d**, **e**) Immunofluorescence analysis of CD34 and DAPI. Objective lens, × 40. (**d**) miPS-LLCcm cells. (**e**) miPS-LLCcm cell-derived primary cells.

To characterize the events induced by cobalt in CSCs, we analyzed the expression of genes and proteins related to the stages of differentiation and stemness in the presence of CoCl_2_. The effect on vascular differentiation was first assessed by the expression of CD31 and VEGFR2 by RT-qPCR. The expression of CD31 and VGFR2 was significantly reduced in the presence of CoCl_2_ compared to that of the untreated cells, while there was no significant difference in VEGFR1, which is considered a marker of hemangioblasts (Fig. 2a, b). In the presence of CoCl_2_, the expression of CD34 and c-KIT, which are hematopoietic cell markers, was found to be increased by flow cytometric analysis (Fig. 2c). The CD34^+^ cells increased during the 5 days up to 46.7% in the presence of CoCl_2_ while the increase was 4.6% without CoCl_2_. The c-KIT level also increased up to 37.7% at Day 5 in the presence of CoCl_2_ while the increase was 33.5% without CoCl_2_. Although the difference was small at Day 5, the differentiation represented by c-KIT appeared faster at Day 3, showing that the c-KIT positive population increased to 12.4% in the presence of CoCl_2_. CD34 expression was further evaluated by immunofluorescence analysis of both miPS-LLCcm cells and their tumor derived primary cultured cells (Fig. 2d,e). CD34 was confirmed to be induced in the presence of CoCl_2_ in both cell lines. Notably, the expression of Runx1, which is responsible for differentiation into mature blood cells, was upregulated in the presence of CoCl_2_ as well as that of CD34 and c-KIT. Cobalt appeared to increase the transcriptional activity of the Runx1 protein related to hematopoietic cell differentiation , which was correlated with HIF-1α expression.

### Cobalt induced cancer stem cells to differentiate into a blood cell phenotype

**Figure 3:**
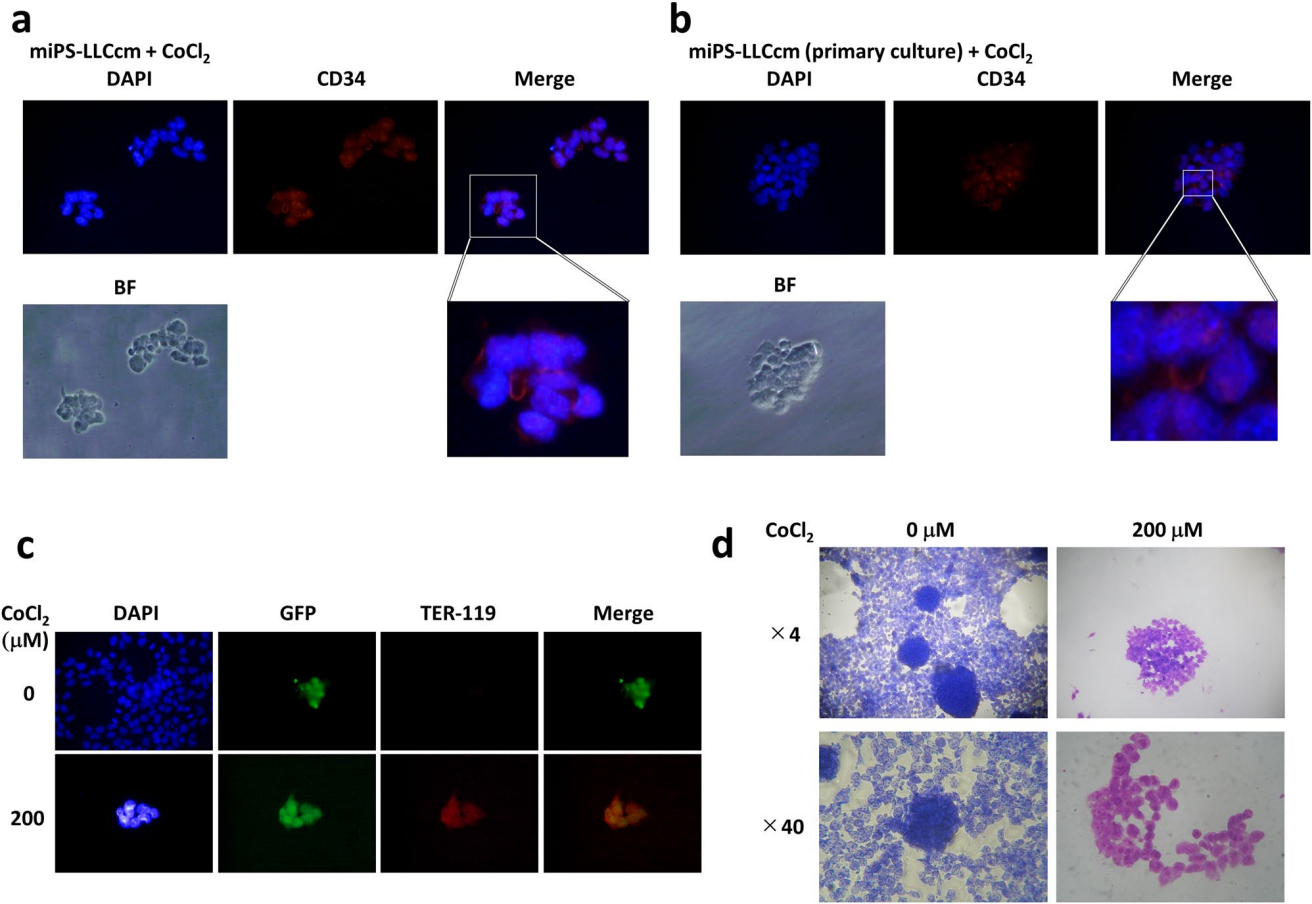
Hematopoietic differentiation of miPS-LLCcm cells in the presence of CoCl_2_. (**a**, **b**) Immunofluorescence analysis of CD34 and DAPI. Squared parts of each image are enlarged to show the absence of DAPI staining surrounded by CD34 positive staining. BF, bright field. Objective lens, × 40. (**a**) miPS-LLCcm cells. (**b**) miPS-LLCcm cell-derived primary cells. (**c**) Immunofluorescence analysis of the erythroblast marker TER-119 together with GFP and DAPI miPS-LLCcm cells. Immunoreactivity to TER-119 was observed in the presence of CoCl_2_. Objective lens, × 40. (**d**) Wright-Giemsa staining of miPS-LLCcm cells. Red coloration was observed in the presence of CoCl_2_.

Further analysis was performed to assess the effect of cobalt on the differentiation of CSCs into a hematopoietic precursor cell phenotype based on the hematopoietic cell phenotype observed at Day 5. On Day 7 of treatment with CoCl_2_, immunostaining showed that the expression of the hematopoietic cell marker CD34 was maintained. In the population of CD34^+^ cells, some cells were found without nuclei when stained with DAPI (Fig. 3a). Similar result was observed in the primary cultured cells derived from the tumor of subcutaneously transplanted miPS-LLCcm cells in the presence of CoCl_2_ (Fig. 3b). The cells without nuclei were considered the result of enucleation, which is a phenomenon observed in the differentiation of erythroblasts into erythrocytes. To confirm the presence of erythroblasts in the presence of CoCl_2_, we evaluated the expression of TER-119, which is a marker of erythroblasts. Immunostaining was positive in the presence of CoCl_2_ compared to that in the untreated cells (Fig. 3c).

Furthermore, the presence of blood cells was confirmed by Wright-Giemsa staining, which is generally used for the detection and analysis of blood type cells. As a result, red to purplish-red color developed in the cells in the presence of CoCl_2_ while blue color developed without CoCl_2_ (Fig. 3d). Collectively, miPS-LLCcm cells have been demonstrated to have the potential to differentiate into erythroblasts and erythrocytes through hematopoietic cells in the presence of CoCl_2_.

### Oligomycin A cancelled the effects of cobalt on the differentiation of CSCs

**Figure 4:**
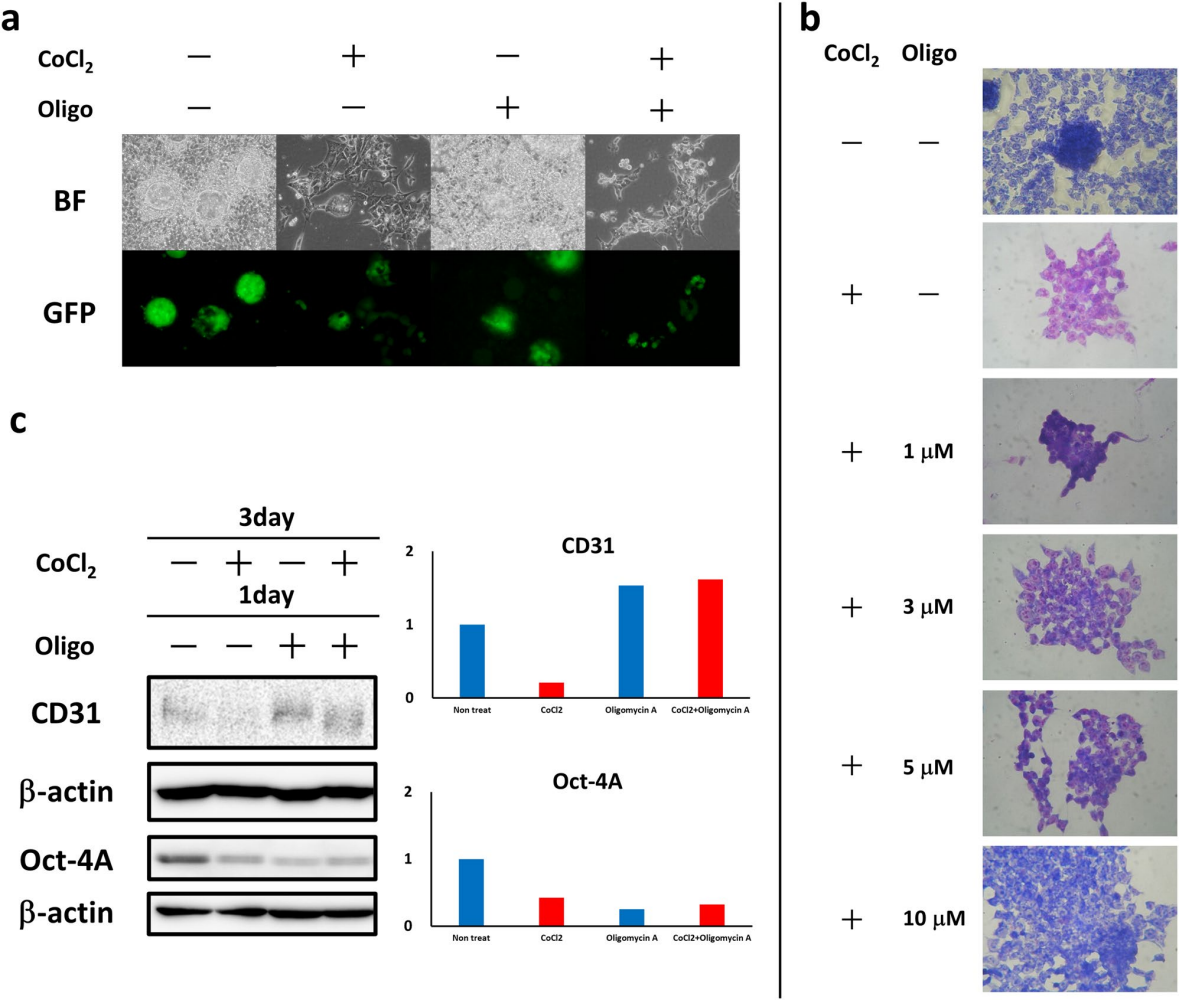
Effect of oligomycin A on miPS-LLCcm cells in the presence of CoCl_2_. (**a**) The effect on morphology and GFP expression was not significant in the presence of oligomycin A. (**b**) Wright-Giemsa staining of miPS-LLCcm cells in the presence of oligomycin A. Red coloration was inhibited by the increase in oligomycin A. (**c**) Western blotting analysis of CD31 and Oct-4A in miPS-LLCcm cells (left). The relative densitometric quantification was performed by ImageJ (right). Oligo, oligomycin A.

miPS-LLCcm cells differentiated into hematopoietic precursor cells in the presence of CoCl_2_. Oligomycin A was previously reported to inhibit the expression of HIF-1α and ATP synthesis via oxidative phosphorylation^23^. In cancer tissues, ATP synthesis is generally considered dependent on glycolysis, as explained by the Warburg effect^24^. In this case, mitochondrial ATP synthesis, which is oxidative phosphorylation, should be relatively low compared to glycolysis. In this context, we thought the effect of CoCl_2_ could be enhanced by oligomycin A inhibiting the respiratory chain in mitochondria. miPS-LLCcm cells were treated with oligomycin A for the last 24 h during the 3 days of CoCl_2_ treatment. As a result, the morphological change of the cells induced by CoCl_2_ was not affected by treatment with oligomycin A (Fig. 4a). In contrast, the red-to-purplish-red color change in Wright-Giemsa staining in the presence of CoCl_2_ was suppressed by oligomycin A in a dose dependent manner. The color change was significantly suppressed in the presence of oligomycin A at 10 µM compared to the blue color without CoCl_2_ (Fig. 4b). For differentiation into hematopoietic progenitor cells, CoCl_2_ enhanced differentiation, and oligomycin A cancelled the effect of CoCl_2_.

The expression of the endothelial cell marker CD31 and the undifferentiated stemness marker Oct-4A was further assessed in the presence or absence of oligomycin A in the presence of CoCl_2_. The results showed that the expression of CD31 and Oct-4A was suppressed, while the expression of CD31 was promoted and the expression of Oct-4A was not affected in the presence of oligomycin A with CoCl_2_ (Fig. 4c). Collectively, cobalt suppressed differentiation into endothelial cells while promoting differentiation into hematopoietic progenitors.

## Discussion

**Figure 5:**
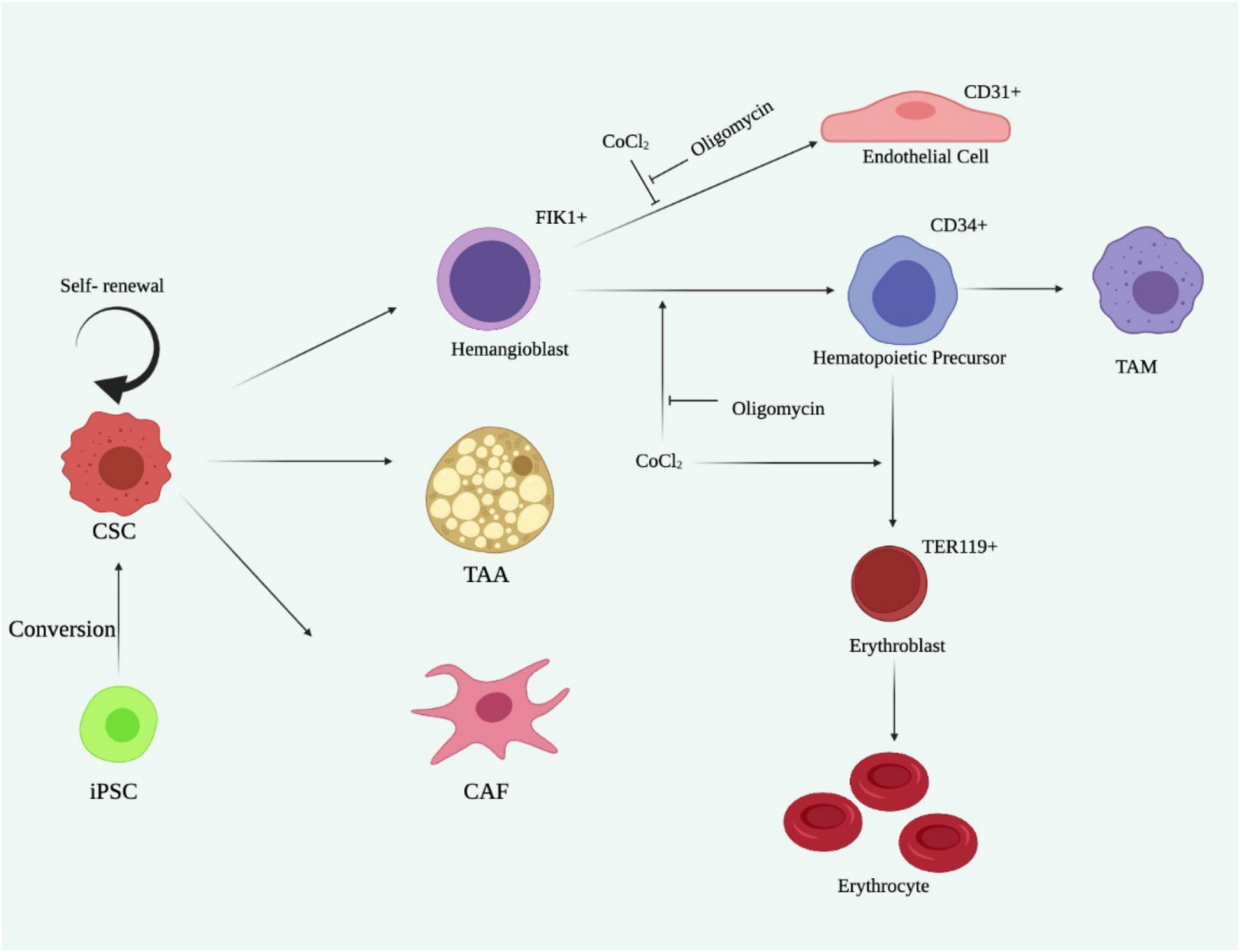
Schematic drawing of cancer stem cells differentiating into cancer associated cell phenotypes. The CSC model was induced from iPSCs^8^ and shown to differentiate into cancer associated fibroblasts (CAFs)^12^, tumor associated adipocytes (TAAs)^27,28^, vascular endothelial cells^14^ including tumor associated macrophages (TAMs)^27,29^. In this study the direction of differentiation was further proposed under hypoxia in TER119^+^ erythroblasts together with enucleation.

Hemangioblasts are generally concentrated in the CD34^+^ fraction^25^. Vascular endothelial progenitor cells are generally considered to be differentiated from hemangioblasts. Progenitor cells will mature into CD31^+^ vascular endothelial cells during angiogenesis. In this context, the differentiation of CSCs into vascular endothelial cells, which we previously demonstrated^14,26^, should occur via the stage of hemangioblasts. From these insights, the possibility of the differentiation of CSCs into blood cells via hematopoietic precursor cells could be hypothesized (Fig. 5).

In this study, we investigated the direction of CSC differentiation in the presence of CoCl_2_. As a chemical element, Co of which atomic number 27 is a metal similar to Fe and Ni. Vitamin B12 (cobalamin) is the primary molecule containing cobalt in mammals^30^ and is a cofactor in methionine synthase and methylmalonyl-CoA mutase supporting methionine/folate synthesis and neoglycogenesis, respectively. In this context, CoCl_2_ may enhance the cell growth and energy metabolism. On the other hand, CoCl_2_ is known to induce chemical hypoxia because it stabilizes HIF-1α and -2α under normoxic conditions. This hypoxia model allows to characterize the molecular and cellular levels of hypoxic response although a decrease of oxygen is the optimal hypoxia model. Fe^2^^+^ is present in the catalytic domain of the hypoxia sensing domain of prolyl hydroxylases, which are the key enzymes that catalyze O_2_ to hydroxylate HIFs under normoxic conditions^22^. Co^2^^+^ can substitute the Fe^2^^+^ in the catalytic domain to inactivate the enzyme activity of prolyl hydroxylation.

Under normoxic conditions, HIFs hydroxylated at proline residues in the oxygen-dependent degradation domain are recognized by the von Hippel-Lindau protein (pVH)^31^, which is a part of the E3 ubiquitin ligase complex, polyubiquitinated and degraded in proteasomes. Co^2^^+^ was also suggested to directly bind to HIF-2α to disrupt the interaction between pVHL and HIF-2α binding within the oxygen-dependent degradation domain^32^. Co^2^^+^ was shown to inhibit the hydroxylation of proline residue within the domain of HIF-2α, to stabilize cytoplasmic HIF-2α occupying the domain binding to pVHL and to inhibit the interaction between pVHL and HIF-2α even HIF-2α was hydroxylated^33^.

Co^2^^+^ was further shown to specifically bind to cullin-2, which is an important component of the E3 ubiquitin ligase complex, which recognized hydroxylated HIF proteins. Although the binding of Co^2^^+^ to cullin-2 did not affect the formation of the ligase complex, some effects on cullin-2 activity were implied^34^.

Under hypoxic conditions, similarly with cobalt, prolyl hydroxylase activity is mainly inhibited, resulting in the increased level of HIF levels. Then HIF-1α/2α translocate to the nucleus, heterodimerizing with HIF-1β and binding to the hypoxia response element to transactivate hypoxia-responsive genes^35^. Under normoxic conditions, the factor inhibiting HIF (FIH), which hydroxylates an asparagine residue in the carboxyl-terminal domain of HIF-1α, disrupts the interaction of HIF-1α with the transcription coactivators p300/CREB-binding protein inhibiting the transcriptional activity^36^. Hypoxia inhibits the asparagine hydroxylation by FIH, allowing the p300/CREB-binding protein complex to bind to HIF-1α/2α and consequently enabling transcription by HIF^37^. Depletion of Fe^+^ inhibited of the activity of both prolyl hydroxylases and FIH, and HIF-1α accumulated exhibiting the activity^38,39^. In this context, the chemical hypoxia induced by CoCl_2_ could apparently mimic the mechanism of hypoxia while the hypoxia is completely opposite to normoxic conditions and Co^2^^+^ may induce cellular proliferation as incorporated into vitamin B12.

Since many cases of differentiation into blood cells have been reported under hypoxic conditions^17–20^, we explored the potential of differentiation into blood cells. We have already demonstrated the potential of differentiation into hematopoietic cells and macrophages with our CSC models induced from iPSCs^27,29^. This kind of differentiation of CSCs will help them survive, supporting their microenvironment in a heterogeneous cell population in tumor tissue as well as tumor angiogenesis, which will provide oxygen and nutrients.

In the presence of CoCl_2_, the morphology of miPS-LLCcm cells exhibiting colonies with sharp edges was altered, and HIF-1α expression increased (Fig. 1). Although GFP^+^ cells remained even with the morphological change to spread with soft edges, differentiation of GFP^-^ cells remarkably increased and colonies appeared to decrease depending on the duration of the treatment with cobalt. When compared with iPSCs, which differentiated in a couple of days, miPS-LLCcm cells could be more resistant to cobalt.

The expression of the hematopoietic stem cell markers VEGFR1 and RUNX1, slightly increased while that of the vascular endothelial cells CD31 and VGFR2, decreased in the presence of CoCl_2_, indicating that endothelial cell differentiation was suppressed but hematopoietic stem cell differentiation was promoted (Fig. 2). Accordingly, the expression of hematopoietic stem cell markers, CD34 and c-KIT, increased depending on the duration of the treatment with cobalt. Collectively, the differentiation into hematopoietic cells is promoted in the presence of CoCl_2_. The enhanced expression of Runx1 may support the promoted differentiation of miPS-LLCcm cells into mature blood cells as well as those of CD34 and c-KIT.

In this context, it was conceivable that the maturation of blood cells was accelerated to produce CD34^+^ cells with enucleation in the CSC model in the presence of CoCl_2_ (Fig. 3). This observation could indicate the differentiation of CSCs into blood cells such as erythroblasts since enucleation is generally considered to occur when these cells differentiate into erythroblasts^40^. The expression of TER-119 and Wright-Giemsa staining supported the finding of the promoted differentiation of CSCs into blood cells in the presence of CoCl_2_.

To assess the effect of CoCl_2_ on the differentiation of CSCs, we evaluated the effect of oligomycin A in the presence of CoCl_2_ (Fig. 4). The morphology was minimally affected, while differentiation was suppressed in the presence of oligomycin A. The expression of CD31 and Oct-4A was inversely correlated, indicating that oligomycin A promoted differentiation. In this context, the morphological effect did not appear to be related to differentiation. Differentiation into hematopoietic stem/blood progenitor cells was suppressed by oligomycin A in the presence of CoCl_2_, as shown by Wright-Giemsa staining. Collectively, cobalt promoted the differentiation of miPS-LLCcm cells into hematopoietic progenitor cells and TER-119^+^ erythroblasts, suppressing their differentiation into endothelial cells.

Taking the function of oligomycin A into consideration, we hypothesized that the hypoxic mimicry with CoCl_2_ was enhanced by oligomycin A. However, the contradictory effects of oligomycin A abolished CSC differentiation by CoCl_2_. This result could be explained by the inhibition of HIF-1α accumulation in hypoxic tumor cells by oligomycin A^23^. Although further investigation is required, the stability of HIF-1α might be significantly responsible for CSC differentiation into hematopoietic progenitors. The genes transcribed by HIF-1α in miPS-LLCcm cells should be clarified in the future.

As summarized in Fig. 5, miPS-LLCcm cells were found to potently differentiate into hemangioblasts, which differentiated not only into vascular endothelial cells^14,26^ but also into hematopoietic progenitor cells leading to macrophages, a type of white blood cell^15^ and erythroblasts, a type of red blood cell, in this study.

## Conclusion

miPS-LLCcm cells exhibited differentiation into endothelial cells and blood progenitor cells. In particular, differentiation into erythroblasts and erythrocytes was promoted in the presence of CoCl_2_ as a mimicry of hypoxia. Oxidative phosphorylation was apparently involved in differentiation into blood cells. These findings are expected to help understand CSC survival, providing progenies that support the hierarchy and heterogeneity of cancer tissues.

## Materials and methods

### Cell culture

miPS-LLCcm cells were prepared as CSCs from miPSCs (iPS-MEF-Ng-20D-17, Lot No. 012, Riken Cell Bank, Tokyo, Japan) by culturing 4 weeks in the presence of the conditioned medium of Lewis lung carcinoma cells^8^. The primary culture of miPS-LLCcm cells were prepared from the malignant tumor developed in a BALB/c nude muse transplanted with miPS-LLCcm cells. miPS-LLCcm cells and miPSCs were maintained in iPS media consisting of DMEM (Sigma-Aldrich, St. Louis, MO, USA), 15% fetal bovine serum (FBS) (Thermo Fisher Scientific), 2 mM L-glutamine (Nacalai Tesque, Kyoto, Japan), 0.1 mM nonessential amino acids (NEAA, Thermo Fisher Scientific) and 0.1 mM 2-mercaptoethanol (Sigma-Aldrich) with 50 U/ml penicillin and 50 µg/ml streptomycin (Wako, Osaka, Japan). For the induction of chemical hypoxia, cells were treated with CoCl_2_ (Sigma-Aldrich) in the range of 0 to 400 µM for 1 to 7 days. During the treatment, medium was changed every day. In addition, cells were treated with oligomycin A (MedChemExpress, NJ, USA) in the range of 0–10 µM to evaluate the relationship with the hypoxic mimicry conditions. Oligomycin A treatment was started on the second day after the treatment with 200 µM CoCl_2_ for 24 h.

### RNA extraction and RT-qPCR

Total RNA was extracted from cells using TRIzol RNA Isolation Reagents (Thermo Fisher Scientific) and RNA was treated with DNase I (Promega, Fitchburg, WI, USA) according to the manufacturer’s instructions. Then, 4 µg of RNA cDNA was synthesized with the GoScript ™ Reverse Transcription System (Promega, Fitchburg, WI, USA) followed by RT-qPCR analysis, which was performed with a Light Cycler 480 II (Roche Diagnostics GmbH, Mannheim, Germany) and Light Cycler 480 SYBR Green I Master Mix (Roche Diagnostics GmbH). The reaction and operation were performed according to the manufacturer’s manual. Gene expression levels were normalized by that of β-actin. The sequences of primers designed to amplify the target genes are as follows: VEGFR1 (GenBank accession No. NM_010228) forward: 5'-TGA CCC ATC GGC AGA CCA ATA C-3', reverse: 5'-AATTCCAGCTCATTTGCACCCTC-3'; VEGFR2 (GenBank accession No. X70842), forward: 5'-TAG GCG CCT GCA CCA AGC CG-3', reverse: 5'-CCT TGC CCT GGC GGA AGC GT-3'; RUNX1 (GenBank accession No. NM_009821.3) forward: 5'-CTG CCC ATC GCT TTC AAG GTG-3', reverse: 5'-CTA TGG TAG GTG GCA ACT TGT GG-3'; CD31 (GenBank accession No. L06039) forward: 5'- AAC TCC TTC ACC ATC AAC AGC ATC-3', reverse: 5'-AAT GAC GTA GCT CTC GGT GTG-3'; β-actin (GenBank accession No. NM_007393) forward: 5'-AAA TCT GGC ACC ACA CCT TC-3', reverse: 5'-GGG GTG TTG AAG GTC TCA AA-3'.

### Western blotting

Under normal conditions, proteins in the lysates of the cells treated with CoCl_2_ and/or oligomycin A were subjected to 8.5 or 12.5% SDS-PAGE. After electrophoresis, the proteins were transferred to PVDF membranes (Immobilon-FL, Merck-Millipore, Cork, Ireland) . The membrane was blocked with 5% skim milk (Snow Brand, Japan) and incubated with the primary antibodies overnight at 4 °C followed by incubation with the suitable secondary antibody. The primary antibodies used were anti-Oct-4A (9B7) mouse monoclonal IgG (1: 1000, #4286, Cell Signaling Technology, MA), anti-HIF-1α mouse monoclonal IgG1 clone # 241,809 (1: 1000, R&D Systems, MN), anti-CD31 rabbit polyclonal antibody (1:500, ab28364, Abcam, UK), anti-mouse β-tubulin rabbit polyclonal antibody (1: 1000, #2146 s, Cell Signaling Technology), anti β-actin mouse monoclonal antibody (1:2000, 010–27,841, Wako) and anti-GFP goat polyclonal antibody labeled with HRP (1: 2000, ab6663, Abcam, UK). The secondary antibodies used were anti-rabbit IgG goat IgG linked with HRP (1:10,000, #7074 s, Cell Signaling Technology)and anti-mouse IgG goat IgG linked with HRP (1: 10,000, #7076 s, Cell Signaling Technology). HRP reaction was performed with Ez West Lumi plus (ATTO, Japan), and the developed fluorescence intensity was detected by Light Capture II (ATTO, Japan) or Lumino Graph I (ATTO, Japan).

### Immunofluorescence

Cells were seeded on gelatin-coated circular micro cover glass (d = 18 mm, Matsunami, Japan) submerged in a 60-mm dish (TPP, Switzerland). After 7 days of treatment with/without 200 µM CoCl_2_, cells were washed with phosphate buffered saline (PBS) 3 times and then fixed with 4% paraformaldehyde (Nacalai-Tesque) at room temperature for 20 min followed by blocking with 10% FBS in PBS containing 0.05% Tween 20 (Nacalai-Tesque) for 1 h at room temperature. The cells were then incubated with anti-CD34 rabbit polyclonal antibody (H-140) (1:50, sc9095, Santa Cruz, CA) or anti-mouse TER-119/erythroid cells rat monoclonal IgG (1:100, 116,201, BioLegend, CA) diluted with blocking solution overnight at 4 °C. After PBS washes, anti-rabbit IgG linked with Alexa Fluor 555 (1:1000, A21427, Invitrogen, CA) was used to detect anti-CD34 antibody and anti-rat IgG linked with Texas red (1:1000, T6392, Invitrogen) was used to detect anti-TER-119 antibody. Incubation with the secondary antibody was performed for 1 h at room temperature. After PBS washes, the samples were prepared on glass slides (Matsunami) using VECTASHIELD Mounting Medium with 4', 6-diamidino-2-phenylindole (DAPI) (Vector Laboratories, CA). Images were taken using an inverted light microscope equipped with a fluorescence light device (IX-80, Olympus, Japan).

### Wright-Giemsa staining

Cells were seeded in a gelatin-coated 60-mm dish by submerging a circular cover glass and treated with or without 200 µM CoCl_2_ for 3 days, followed by treatment with oligomycin A in the range of 0 to 10 µM for 24 h. The cells were washed with PBS and fixed with methanol (Wako) for 5 min at room temperature and then stained with Wright Giemsa stain I (MUTO, Japan) at room temperature for 10 min. After washes with water, the cover glass was air-dried and fixed onto slide glass using a Soft Mount (Wako). Images of cells were observed under a biological microscope (DPTIPHOT, Nikon, Japan) and photographs were taken with a digital camera.

### FACS analysis

Cells were seeded in a gelatin-coated 60-mm dish. After 3 and 5 days of treatment with/without 200 µM CoCl_2_, the cells were trypsinized and washed 3 times with PBS. Then, the cells were stained with anti-CD34 rabbit antibody (1:167) or anti-CD117/c-KIT rabbit antibody (1:167, #3074 T Cell Signaling Technology) for 30 min at room temperature. After incubation, the cells were washed and incubated with anti-rabbit IgG goat antibody linked with Alexa Fluor 647(1:1000, #4414S Cell Signaling Technology). The cells were analyzed on an Accuri C6 Plus flow cytometer (BD Bioscience, San Jose, CA) and then analyzed by FlowJo software excluding the patterns of cell debris and aggregates based on scatter signals.

### Statistical analysis

The data presented in this study were taken from three independent experiments and are depicted as the mean ± SD. Statistical comparisons between experimental groups were analyzed by t-tests and *p* < 0.05 was considered statistically significant.

## Supplementary Information

Supplementary Information.

## Data Availability

All data generated and/or analyzed during this study are included in this article.
