# Supplementary material for: Differentiation of cancer stem cells into erythroblasts in the presence of CoCl2
Source: Sci Rep. 2021 Dec 14;11:23977. doi: 10.1038/s41598-021-03298-5 (PMC8671479; doi:10.1038/s41598-021-03298-5)

Whole view of Fig. 2b (left)

Oct-4A

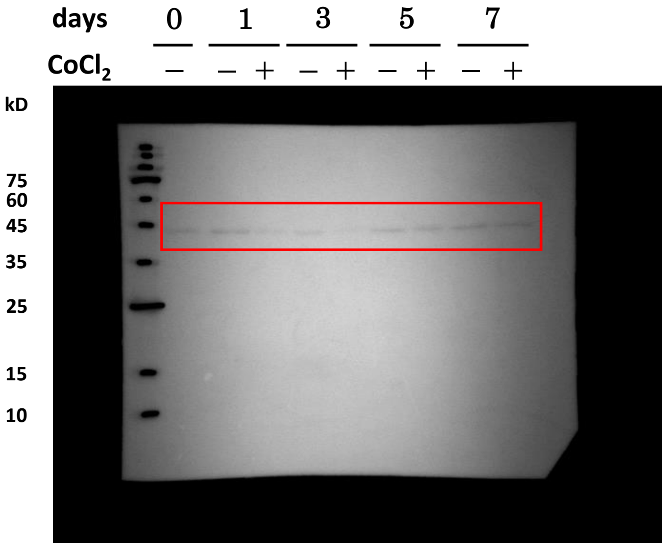

GFP

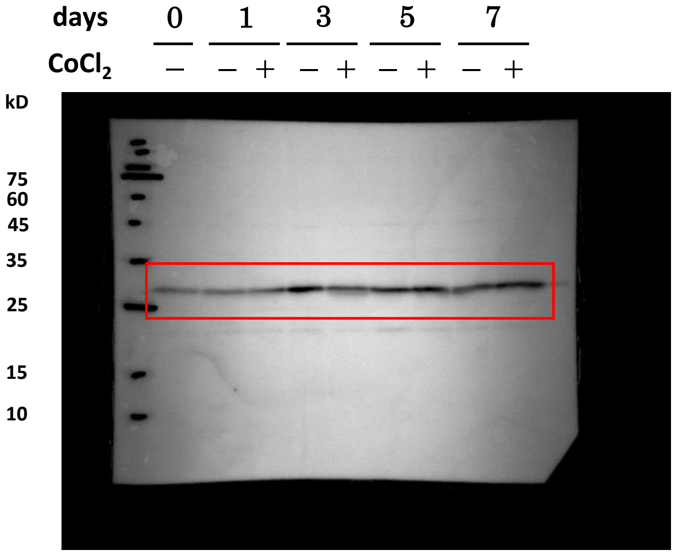

HIF-1α

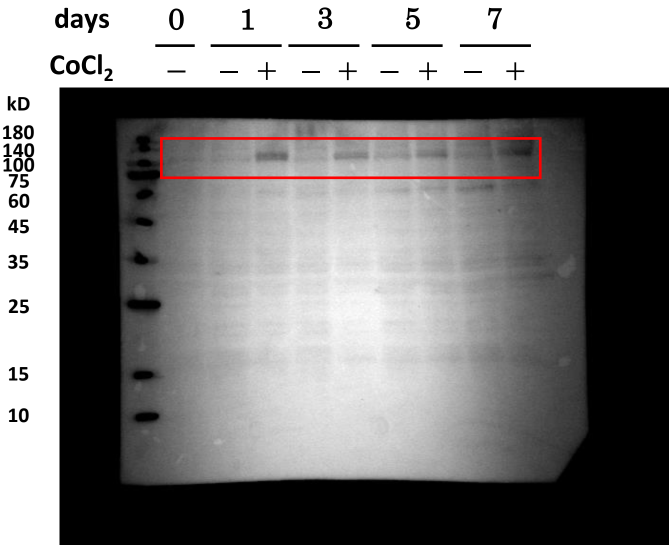

β-tubulin

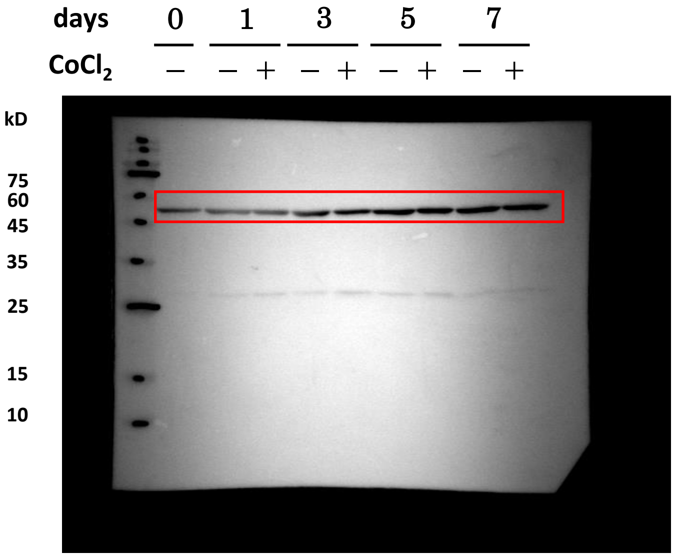

General view of Fig. 2b (right)

CD31

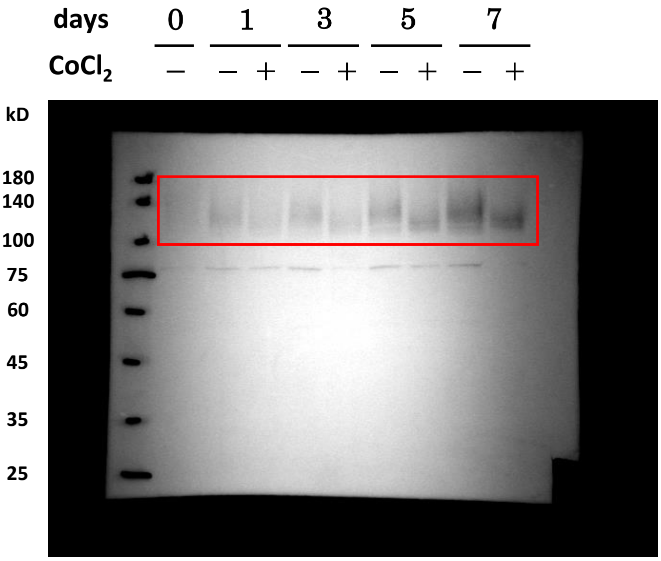

β-tubulin

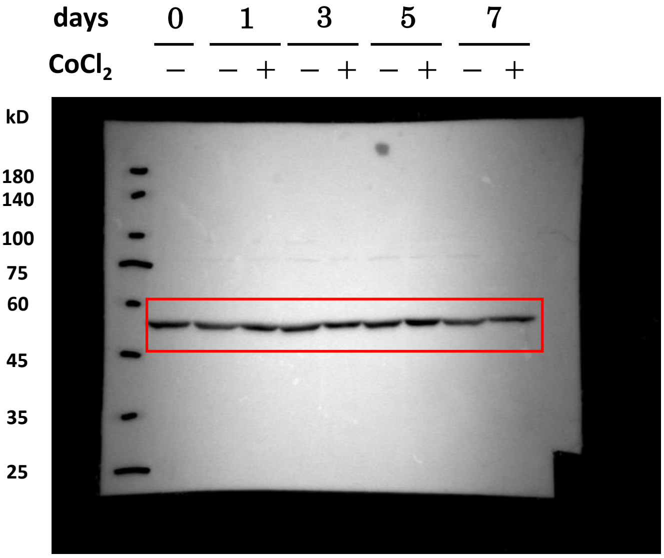

Whole view of Fig. 4c

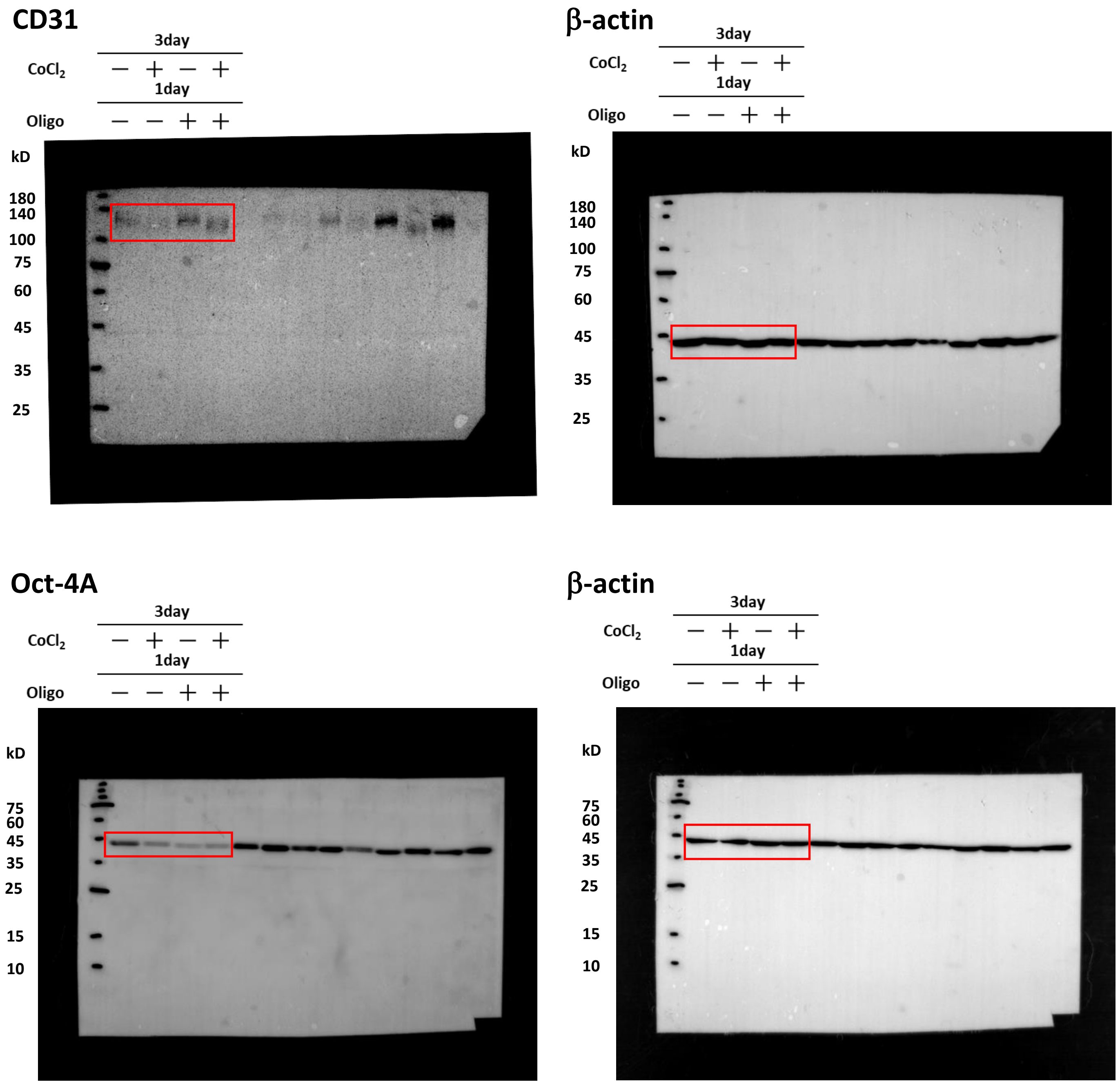

Supplement: Supplementary file 1 — Supplementary Information. [file 41598_2021_3298_MOESM1_ESM.pdf]
